# A Novel Homozygous *ATP7B* Splice-Site Variant Causes Late-Onset Hepatic Wilson Disease

**DOI:** 10.14309/crj.0000000000002256

**Published:** 2026-08-21

**Authors:** Ouwais Alkhateb, Don C. Rockey, Kassem Barada

**Affiliations:** 1 Department of Internal Medicine, American University of Beirut, Beirut, Lebanon; 2 Digestive Disease Research Center, Medical University of South Carolina, Charleston, SC; 3 Division of Gastroenterology and Hepatology, American University of Beirut, Beirut, Lebanon

**Keywords:** cryptogenic cirrhosis, lean MASH, Wilson disease, *ATP7B* mutations

## Abstract

Wilson disease (WD) is a rare disorder that may be missed because of atypical presentations. We present a 47-year-old asymptomatic man who was incidentally discovered to have hepatic steatosis and cirrhosis without features of the metabolic syndrome and with normal physical examination and liver function tests. Very low ceruloplasmin and high urine copper prompted evaluation for WD. He had no Kayser–Fleischer rings. Genetic testing revealed a novel homozygous splice-site *ATP7B* variant (NM_000053.2:c.1707+2dupT p.(?)), the first reported case of homozygosity for this mutation. This case highlights the importance of considering WD in patients with unexplained hepatic steatosis and cryptogenic cirrhosis.

## INTRODUCTION

Wilson disease (WD) is a rare autosomal recessive disorder of copper metabolism caused by mutations in the *ATP7B* gene. WD can mimic metabolic dysfunction-associated steatotic liver disease (MASLD), particularly in lean individuals.^1^ Hepatic involvement is common, and early liver pathology often includes steatosis, which may be indistinguishable from MASLD.^1–3^

We report a 47-year-old asymptomatic man with cryptogenic cirrhosis and “fatty liver” who was initially presumed to have lean MASLD but was ultimately diagnosed with WD. The patient had very low serum ceruloplasmin, high urine copper, normal brain magnetic resonance imaging (MRI), and no Kayser–Fleischer (KF) rings. Genetic testing revealed a novel homozygous splice-site variant (c.1707+2dupT). This *ATP7B* variant had been reported previously only in the heterozygous state, making this the first described homozygous case.^4^

## CASE REPORT

**Figure 1.**
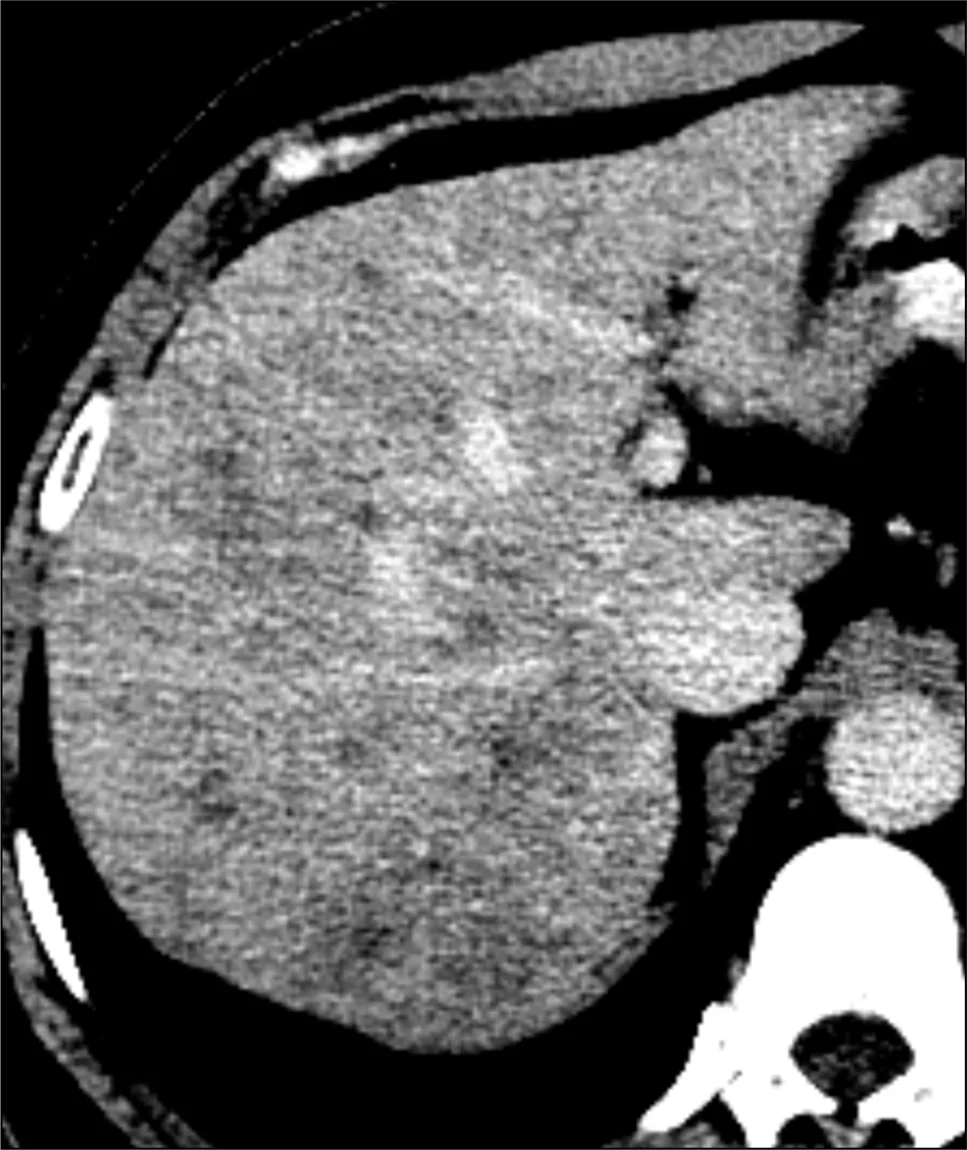
Contrast-enhanced axial CT images of the abdomen showing diffuse heterogeneous hepatic parenchyma with numerous small hypodense hypoenhancing nodules that led to further evaluation by MRI. CT, computed tomography.

A 47-year-old man with a history of nephrolithiasis and hypertension presented in August 2024 with 2 days of right flank pain. Computed imaging revealed heterogeneous hepatic parenchyma with numerous small hypodense nodules (Figure 1). Subsequent MRI revealed diffuse heterogeneous fatty infiltration of the liver, multiple small hepatic nodules, and nodular hepatic contour, suggestive of cirrhosis. He was then referred to a gastroenterologist.

The patient denied alcohol intake. His only regular medication was telmisartan plus hydrochlorothiazide. Physical examination showed a body mass index of 25 kg/m^2^ and a nodular liver. Besides his controlled hypertension, he had no other evident risk factors of metabolic syndrome. His laboratory workup revealed normal fasting glucose, lipid profile, liver enzymes, and liver function tests. Viral and autoimmune hepatitis workup was negative (Table 1).

**Table 1.** Summary of the diagnostic evaluation performed for the reported patient

Investigation category	Test	Result
Metabolic evaluation	Fasting glucose	89 mg/dL
	Triglycerides	117 mg/dL
	LDL cholesterol	65 mg/dL
	HDL cholesterol	70 mg/dL
Viral hepatitis workup	HBsAg	Negative
	HCV total antibodies	Negative
Autoimmune liver disease workup	ANA	Negative
	Anti-dsDNA IgG	Negative
	AMA-M2 antibodies	Negative
	Liver-kidney microsomal type 1 antibodies	Negative
	Smooth muscle antibodies	Negative
	Serum IgG	13.42 g/L (normal)
	Serum IgA	3.08 g/L (normal)
Celiac disease screening	Tissue transglutaminase IgA	Normal
Iron overload evaluation	Ferritin	700 ng/mL
	Transferrin saturation	37.9% (normal)
	HFE mutation analysis	Negative

AMA, antimitochondrial antibody; ANA, antinuclear antibody; HBsAg, hepatitis B surface antigen; HCV, hepatitis C virus; HDL, high-density lipoprotein; HFE, high Fe (iron) gene; IgA, immunoglobulin A; IgG, immunoglobulin G; LDL, low-density lipoprotein.

The patient was completely asymptomatic and had no family history of any liver disease. Transient elastography in September 2024 showed a controlled attenuation parameter score of 292 dB/m and liver stiffness measurement (LSM) of 12.3 kPa, consistent with steatosis and advanced liver fibrosis. Autoimmune serologies were negative. His serum ceruloplasmin was markedly low <3 mg/dL (reference range 15–30 mg/dL), and his 24-hour urine copper was 80 µg/24 hr (reference range <50 µg/24 hr).

The patient lost 10 kg while trying to treat his fatty liver. A repeat LSM in December 2024 showed an increase in LSM to 15.6 kPa with resolution of liver steatosis (S0, controlled attenuation parameter score 238 dB/m). Examination with slit lamp revealed no KF rings. Neurologic examination and brain MRI were normal. His plasma copper level was 20 µg/dL (reference range 70–130 µg/dL), and spot urine copper was 36 µg/dL (reference range <2 µg/dL). Ferritin was elevated at 700 ng/mL, with normal iron % saturation. High Fe (iron) gene mutation analysis was negative. A repeat serum ceruloplasmin was still low at <3 mg/dL. A diagnosis of lean metabolic dysfunction-associated steatohepatitis was contemplated.

The patient has 2 daughters, including a 6-month-old infant who was being investigated for macrocephaly, hypotonia, and failure to thrive. Concerned about his low ceruloplasmin and for his daughter's health, the patient requested genetic testing for WD on himself. Testing for the *ATP7B* gene revealed that he was homozygous for *ATP7B* NM_000053.2: c.1707+2dupT p.(?), with a class 2 (likely pathogenic) splicing variant. This confirmed WD, making a liver biopsy unnecessary. The patient was referred for genetic counseling to screen his siblings and both of his daughters. Treatment with a low copper diet and trientine hydrochloride was initiated. Three weeks into treatment, a 24-hour urine copper level was 833 µg/24 hr, above the target of >500 µg/24 hr.

## DISCUSSION

WD, an autosomal recessive disorder of copper metabolism caused by mutations in the *ATP7B* gene, classically manifests in children or adolescents with hepatic, neurologic, or psychiatric symptoms.^1^ However, up to 8% of patients present after the of age 40 years, while 40%–60% have hepatic involvement as their first clinical manifestation.^1,2^ This patient's age (47 years), absence of KF rings, and lack of neuropsychiatric symptoms initially obscured the diagnosis, emphasizing that WD should remain in the differential diagnosis even in middle-aged individuals with unexplained liver disease.

WD may be misdiagnosed as MASLD because it may present with prominent steatosis.^2^ Indeed, a diagnosis of lean metabolic dysfunction-associated steatohepatitis was contemplated in our patient. However, histological steatosis is observed in over 50% of patients with WD and represents an early hepatic manifestation of WD that may be histologically indistinguishable from MASLD.^2,3^ For this reason, WD may be misdiagnosed as metabolic steatohepatitis, particularly in lean individuals, as occurred in our patient. Although 7%–20% of patients with MASLD are lean, the finding of hepatic steatosis in such patients should prompt evaluation of alternative etiologies of liver disease.^5^ These include genetic conditions such as WD, α-1 antitrypsin deficiency, autoimmune processes such as celiac disease, and endocrine disorders such as hypothyroidism.

Accordingly, current clinical practice guidelines recommend a stepwise evaluation for alternative diagnoses in lean individuals with suspected MASLD, with testing for WD primarily based on expert opinion.^5,6^ Our case contributes to the growing evidence supporting testing of WD in this population.

**Figure 2.**
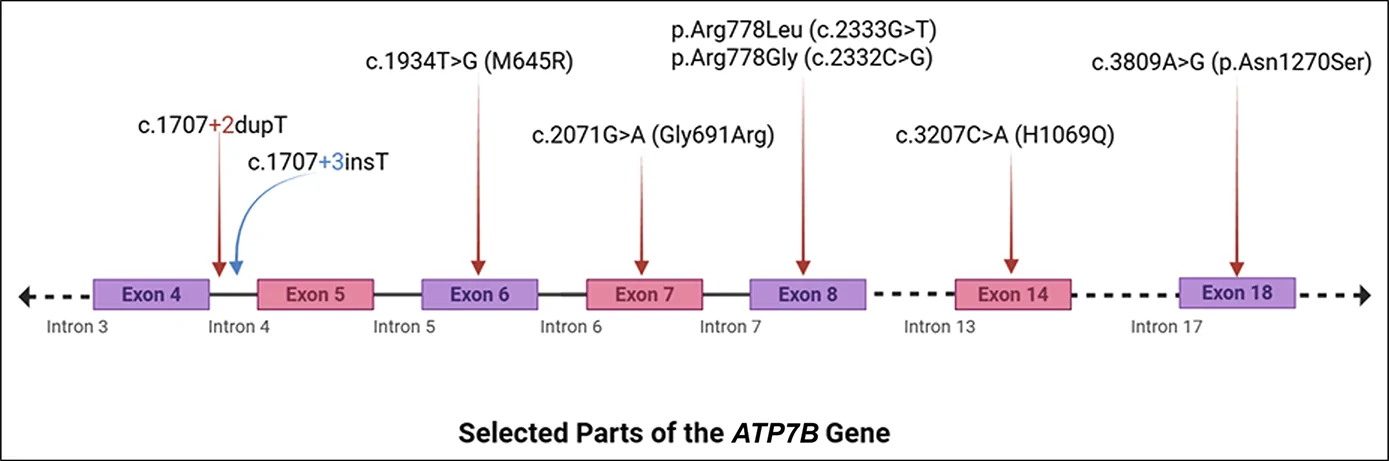
Schematic representation of selected *ATP7B* gene mutations reported in Wilson disease, including the intron 4 splice-site mutation c.1707+2dupT identified in our patient. Colored rectangles represent exons, while lines in between them represent introns.

In addition, current guidelines recommend genetic testing for WD after equivocal conventional testing when clinical suspicion persists.^7^ In Lebanon, where our patient is from, the 2 most common mutations are missense mutations Gly691Arg (c.2071G>A) and p.Asn1270Ser (c.3809A>G), accounting for approximately 27% and 22%, respectively (Figure 2).^8^ These mutations occur within coding exons and lead to amino acid substitutions in the ATP7B protein—disrupting its function through structural changes in critical domains such as the transmembrane regions and ATP-binding sites. By contrast, genetic testing in our patient identified a rare splice-site homozygous mutation in the *ATP7B* gene, designated NM_000053.2: c.1707+2dupT p.(?), classified as a class 2 likely pathogenic mutation. This variant identified in our patient is located in intron 4, where it disrupts the canonical splice donor site. It is predicted to cause aberrant messenger ribonucleic acid splicing, potentially resulting in a frameshift or premature stop codon, and thus a nonfunctional, dysfunctional, or truncated protein.^8–11^

In general, splice-site mutations in *ATP7B* disrupt normal pre-messenger ribonucleic acid processing and could result in exon skipping or activation of cryptic splice sites. This could introduce frameshifts and premature termination codons, which could generate abnormal transcripts and truncated or functionless protein. Because of that, splice-site mutations often display an earlier and more severe phenotypic presentation, which is typically hepatic. Nevertheless, this is not always predictable, as residual *ATP7B* activity can persist in different organs.^10,12,13^ This could be the case in our patient, who had a delayed presentation with asymptomatic liver cirrhosis. Nonetheless, it would be prudent to monitor early and frequently liver function in affected patients.

To date, the splice-site mutation found in our patient has been reported only in 3 cases worldwide, all in the compound heterozygous state.^9–11^ Our patient represents the first reported case of homozygosity for this variant.^4^ Notably, previously published cases demonstrated only hepatic involvement, without neurologic or ocular involvement (Table 2). One case involved an asymptomatic child diagnosed at the age of 8 years, while 2 others presented with acute liver failure at the age of 44 and 58 years, respectively.^9–11^ In addition, splice-site mutations have been associated with a younger onset presentation and with fewer neurologic manifestations than missense-only genotypes.^14^ These observations suggest a potential genotype–phenotype correlation unique to this specific splice-site variant, although further cases are needed to validate this association. This case further supports the reclassification of this variant from “likely pathogenic” to “pathogenic,” as WD diagnosis was confirmed even before genetic testing according to the modified Leipzig scoring system (Table 3), with the patient meeting the established diagnostic criteria (score ≥4).^3^

**Table 2.** Characteristics of patients with confirmed Wilson disease who have the c.1707+2dupT mutation—literature review

	Alkhateb et al (in press)	Loudianos et al^10^	Németh et al^11^	Weitzman et al^9^
Mutation	c.1707+2dupT	c.1707+3insT	c.1707+2dupT	c.1707+3insT
Zygosity	Homozygous	Compound heterozygous	Compound heterozygous	Compound heterozygous
Place of origin	Middle East	Greek	Hungary	Unknown
Age at diagnosis	47	8	44	58
Neurologic	Absent	Absent	Absent	Absent
Kayser–Fleischer Ring	Absent	Absent	Absent	Absent
Phenotype	Asymptomatic	Asymptomatic	Acute liver failure	Fulminant liver failure

**Table 3.** Modified Leipzig scoring system for Wilson disease

Parameter	Score
Kayser–Fleischer rings	
Present	2
**Absent**	**0**
Serum ceruloplasmin	
>20 mg/dL (normal)	0
**0–5 mg/dL**	**3**
6–11 mg/dL	2
11–20 mg/dL	1
24 h urinary copper (in the absence of chronic cholestatic liver disease)	
>100 µg	2
**40–100 µg**	**1**
<40 µg	0
Coombs-negative hemolytic anemia with liver disease	
Present	1
Absent	0
Mutational analysis	
**On both chromosomes detected**	**4**
On one chromosome detected	1
No mutation detected/test not done	0
Liver biopsy for histology suggestive of Wilson disease with	
Orcein- or rhodamine-positive granules	1
Neurobehavioral symptoms	
Present	2
**Absent**	**1**
Typical features on magnetic resonance imaging brain	
Present	1
**Absent**	**0**
History of Wilson disease in a family member	
Sibling death from liver disease/neurological disease suggestive of Wilson disease	1
Evaluation	Total score
**Diagnosis highly likely/established**	**≥4**
Diagnosis possible, more tests needed	3
Diagnosis very unlikely	≤2

Patient-specific values are shown in bold.

This case suggests that patients with lean MASLD need workup to exclude other causes of liver disease, particularly WD. Both groups may have fatty liver, and both may progress to cirrhosis. Hence, WD may be missed if not looked for. Furthermore, patients with WD may have asymptomatic liver disease including cirrhosis, even when they reach middle age.

Early diagnosis allows pre-emptive treatment to prevent further copper damage, as untreated WD is invariably fatal.^7,11^ Current American Association for the Study of Liver Diseases (AASLD) guidelines recommend lifelong chelation therapy (eg, trientine, D-penicillamine) or zinc salts for WD, with trientine preferred due to its favorable side-effect profile, as was begun in our patient.^7^

## DISCLOSURES

Author contributions: O. Alkhateb: Primary author; responsible for drafting and writing the manuscript. DC Rockey: Provided expert input and overall oversight of the manuscript. K. Barada: Contributed to manuscript editing, provided supervisory oversight, and is the article guarantor.

Financial disclosure: None to report.

Informed consent was obtained for this case report.

## ABBREVIATIONS:

AMA: antimitochondrial antibody
ANA: antinuclear antibody
ATP7B: ATPase copper transporting beta
DNA: deoxyribonucleic acid
HBsAg: hepatitis B surface antigen
HCV: hepatitis C virus
HDL: high-density lipoprotein
HFE: high Fe (iron) gene
IgA: immunoglobulin A
IgG: immunoglobulin G
KF: Kayser–Fleischer
LDL: low-density lipoprotein
LSM: liver stiffness measurement
MASH: metabolic dysfunction-associated steatohepatitis
MASLD: metabolic dysfunction-associated steatotic liver disease
MRI: magnetic resonance imaging
WD: Wilson disease
